# Conformations and molecular interactions of poly-γ-glutamic acid as a soluble microbial product in aqueous solutions

**DOI:** 10.1038/s41598-017-13152-2

**Published:** 2017-10-06

**Authors:** Ling-Ling Wang, Jian-Tao Chen, Long-Fei Wang, Sha Wu, Guang-zhao Zhang, Han-Qing Yu, Xiao-dong Ye, Qing-Shan Shi

**Affiliations:** 1 State Key Laboratory of Applied Microbiology Southern China, Guangdong Provincial Key Laboratory of Microbial Culture Collection and Application, Guangdong Open Laboratory of Applied Microbiology, Guangdong Institute of Microbiology, Guangzhou, 510070 China; 2 Faculty of Materials Science and Engineering, South China University of Technology, Guangzhou, 510640 China; 3 Department of Chemistry, University of Science and Technology of China, Hefei, 230026 China; 4 Hefei National Laboratory for Physical Sciences at the Microscale, Department of Chemical Physics, University of Science and Technology of China, Hefei, 230026 China

## Abstract

Soluble microbial products (SMPs) are of significant concern in the natural environment and in engineered systems. In this work, poly-γ-glutamic acid (γ-PGA), which is predominantly produced by *Bacillus* sp., was investigated in terms of pH-induced conformational changes and molecular interactions in aqueous solutions; accordingly, its sedimentation coefficient distribution and viscosity were also elucidated. Experimental results indicate that pH has a significant impact on the structure and molecular interactions of γ-PGA. The conformation of the γ-PGA acid form (γ-PGA-H) is rod-like while that of the γ-PGA sodium form (γ-PGA-Na) is sphere-like. The transformation from α-helix to random coil in the γ-PGA secondary structure is primarily responsible for this shape variation. The intramolecular hydrogen bonds in the γ-PGA-H structure decrease and intramolecular electrostatic repulsion increases as pH increases; however, the sedimentation coefficient distributions of γ-PGA are dependent on intermolecular interactions rather than intramolecular interactions. Concentration has a more substantial effect on intermolecular electrostatic repulsion and chain entanglement at higher pH values. Consequently, the sedimentation coefficient distributions of γ-PGA shift significantly at pH 8.9 from 0.1 to 1.0 g/L, and the viscosity of γ-PGA (5% w/v) significantly increases as pH increases from 2.3 to 6.0.

## Introduction

Soluble microbial products (SMPs) secreted by microorganisms in the natural environment and in engineered systems are important and have been extensively studied in the fields of microbiology, geochemistry, biological wastewater treatment and biological fermentation^1–3^. They are constituents of natural organic matter, which is ubiquitous in water sources, and is a result of interactions between the hydrologic cycle, biosphere and geosphere^4^. In addition, SMPs are especially important in biological wastewater treatment systems because they often form a large portion of the soluble organic material in the effluent and cause membrane fouling^5,6^. Some SMPs, such as poly-γ-glutamic acid (γ-PGA), are commercially produced and used in many industries, including food, pharmaceuticals, healthcare, and water treatment^7^.

SMP characteristics can significantly affect their adsorption, stabilization/aggregation, dissolution and surface transformation in environmental processes and engineered systems^8,9^. Although clearly significant in the natural environment and engineered systems, better characterization of SMPs has proven to be challenging because the complexity of their constituents results in highly variable chemical structures, functional groups, molecular weight distribution, conformations, and surface charge^10^. Previous SMP studies have primarily focused on their origin, formation, and their various compounds, as well as their overall properties such as content, functional groups, molecular weight distribution, and hydrophobic and hydrophilic properties^10,11^. However, the relationships between their constituents and characteristics remain unclear. There is considerable more analytical work required to fully understand the characteristics of SMPs. Several studies have investigated various SMPs that have a regular composition and pattern, such as xanthan and γ-PGA, to reveal the relationship between their conformational changes and metal binding properties^12,13^.

γ-PGA is an SMP predominantly produced and secreted into the natural environment by water and soil bacteria belonging to the genus *Bacillus* sp., such as *B. licheniformis*, *B. subtilis*, *B. megaterium*, *B. pumilis*, *B. mojavensis*, *B. amyloliquefaciens*, and *B. anthracis* ^13^. Some of the physiological functions carried out by γ-PGA include sequestration of toxic metal ions, and resistance to dewatering, nutrient shortage, and antibodies^7,13^. Additionally, γ-PGA has high water solubility, viscosity, biodegradability, biocompatibility, and is non-toxic towards human and the environment. Furthermore, this compound has potentially wide applications in the fields of medicine, cosmetics, foods, water treatment, and agriculture^14,15^.

Several investigations concerning the conformation of γ-PGA have been undertaken, mainly using circular dichroism (CD) measurements and infrared spectra^16,17^. Results have revealed that the unionized polymer takes on a helical conformation, while the ionized polymer behaves as a random coil. The intramolecular hydrogen bonds are considered the major force for the helical conformation; however, the conformational changes and molecular interactions, including intramolecular and intermolecular interactions of γ-PGA at different concentrations in aqueous solutions, have not been completely elucidated.

In dilute solutions, γ-PGA molecules are sequestered from each other and behave independently; the polymer chains interact primarily with the solvent molecules. Conversely, at concentrations, the polymer chains overlap and become entangled^18^; thus, the intermolecular interactions are greatly increased when compared to the chains in dilute solutions. The highest yield of γ-PGA secreted into culture medium has been reported as an extremely high value of 73.0 g/L^19^. Thus, the effect of concentration on conformational changes and intramolecular and intermolecular interactions of γ-PGA could not be excluded in practical conditions, especially during the processes of fermentation, separation and purification of γ-PGA.

Therefore, the main objective of our study is to explore the properties of γ-PGA, an SMP produced by *B. licheniformis*, especially with respect to its conformations, as mediated by pH, and its intramolecular and intermolecular interactions in different concentrations. To that end, we interpreted the conformational-relevant properties (sedimentation coefficient distributions and viscosity) using a combination of Analytical Ultracentrifugation (AUC), Atomic Force Microscopy (AFM), Circular Dichroism (CD), Laser Light Scattering (LLS), and Fourier Transform Infrared Spectroscopy (FTIR) techniques. Applying these techniques can aid in a greater fundamental understanding of the characteristics and roles of γ-PGA (or structurally similar polymers) that are found in the natural environment and in the fermentation industry. Furthermore, this research can be applied in the fields of medicine, cosmetics, foods, water treatment, and agriculture.

## Results

### Effect of pH on γ-PGA viscosity

**Figure 1:**
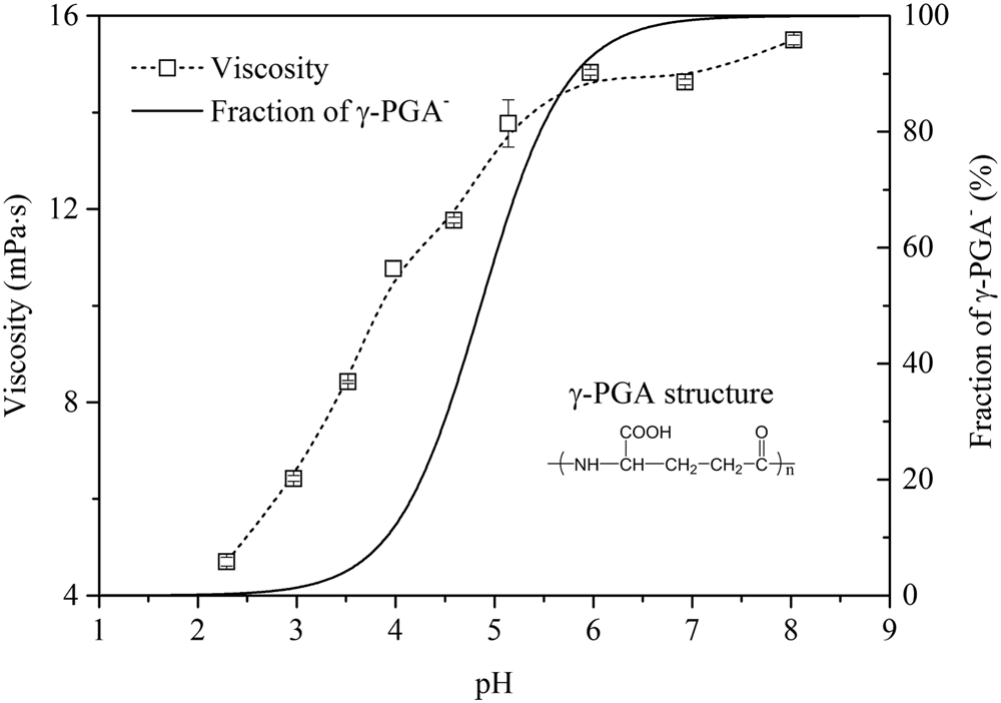
Effect of pH on the viscosity of a γ-PGA solution (5% w/v) and a γ-PGA^−^ fraction.

The effect of pH on the viscosity of the γ-PGA solution and the γ-PGA^−^ fraction was investigated (Fig. 1). The viscosity of the γ-PGA solution appeared to be highly related to the deprotonated degree of the COOH groups in the γ-PGA side chains. The viscosity increased significantly from 4.7 ± 0.1 to 14.8 ± 0.1 mPa·s as the pH increased from 2.3 to 6.0, after which the viscosity slightly increased from pH 6.0 to 8.0. Meanwhile, the acid form of γ-PGA-H rapidly ionized to form the mono-anionic γ-PGA^−^ species as the pH increased from 3.0 to 6.0, while the viscosity of the γ-PGA^−^ species slightly increased with an increase in pH from 6.0 to 8.0.

### Sedimentation coefficient distributions

**Figure 2:**
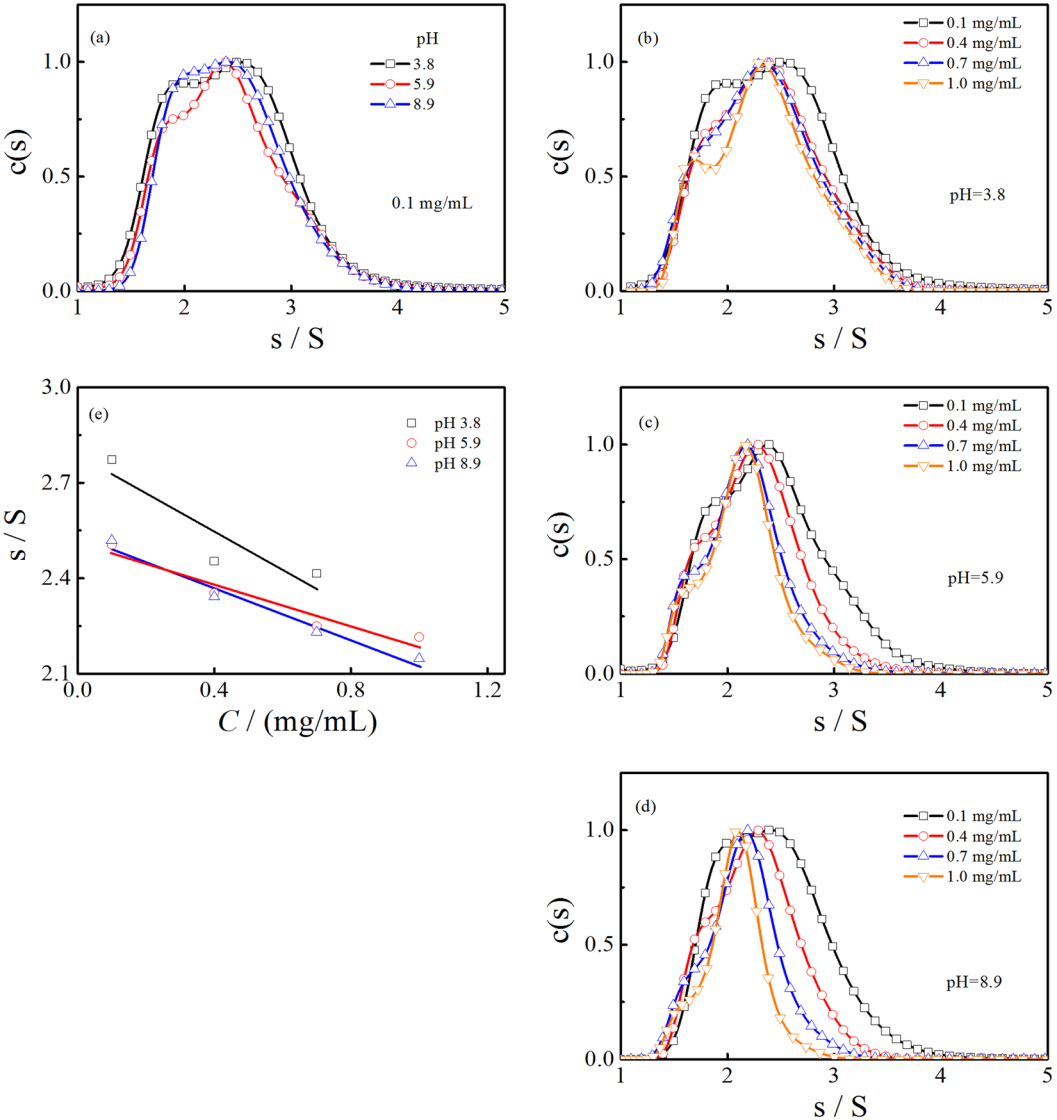
Sedimentation coefficient distributions of a 0.1 g/L γ-PGA at pH 3.8, 5.9, and 8.9 (**a**). Sedimentation coefficient distributions of γ-PGA from 0.1 to 1.0 g/L at pH 3.8 (**b**), pH 5.9 (**c**), and pH 8.9 (**d**). Concentration dependence of the γ-PGA sedimentation coefficient at different pH values (**e**). The *c*(*s*) distributions are normalized to the peak height and *s* represents Svedberg (S) units or 10^−13^ sec.

The sedimentation coefficient distributions of γ-PGA at various concentrations (at pH 3.8, 5.9, and 8.9) were analyzed using the SEDFIT program. The continuous *c*(*s*) distribution model in the SEDFIT program can distinguish boundary spreading due to size heterogeneity from diffusion^20^. The sedimentation coefficient of dilute γ-PGA solutions (0.1 g/L) at pH 3.8, 5.9, and 8.9 are depicted in Fig. 2(a). The naturally occurring γ-PGA (0.1 g/L) presented broad sedimentation coefficient distributions at pH 3.8, 5.9, and 8.9 (Fig. 2(a)). The range of the sedimentation coefficient, *s*, which is commonly referred to in Svedberg (S) units and represents 10^−13^ seconds, varied from approximately 1.3 to 4.0 S, with peaks at 2.5, 2.4, and 2.4 S at pH 3.8, 5.9, and 8.9, respectively.

The partial specific volume of the γ-PGA solution was determined to be 0.656, 0.715, and 0.730 mL/g at pH 3.8, 5.9, and 8.9, respectively. The variation of the partial specific volume with pH is significant and suggests that large differences in solvation is associated with pH. Two factors contribute to the partial specific volume; one is the intrinsic volume of the added solute and the other is the change in volume as a result of the interaction between the hydrated shell and the solute. As pH values increase, the conformational change and electrostatic interactions promote the binding of water molecules and ions to the carboxylated groups.

As the γ-PGA concentration increased, the peaks and distribution of the sedimentation coefficient varied at pH 3.8, 5.9, and 8.9. The effects of γ-PGA concentrations on the sedimentation coefficient distributions were more remarkable at higher pH values. At a lower pH (pH 3.8), the broad sedimentation coefficient distributions at 0.1 g/L γ-PGA were slightly narrow at 0.4, 0.7, and 1.0 g/L, with the peaks shifted to approximately 2.4, 2.3, and 2.3 S, respectively (Fig. 2(b)). At pH 5.9, the width of the sedimentation coefficient distribution reduced gradually as the γ-PGA concentrations increased. The peaks for the 0.4, 0.7, and 1.0 g/L γ-PGA at pH 5.9 shifted to approximately 2.3, 2.2, and 2.1 S, respectively (Fig. 2(c)). At a higher pH (pH 8.9), the areas of the sedimentation coefficient distributions were reduced as the concentration increased; the peaks at 0.4, 0.7, and 1.0 g/L γ-PGA shifted to approximately 2.3, 2.2, and 2.1 S, respectively (Fig. 2(d)).

Multi-peak distributions can be seen under the various experimental conditions (Fig. 2), especially in low pH solutions, indicating that multiple species exist in the solution. As discussed below, γ-PGA appears as rod-like shapes in an acid solution. Furthermore, small and large particles coexist; however, when the rod-like γ-PGA turns to sphere-like shapes, the size difference becomes negligible. Consequently, the *c*(*s*) distribution becomes essentially unimodal with increasing pH.

Figure 2(e) demonstrates the concentration dependence of *s*. In dilute solutions, the weak interactions of the diffuse γ-PGA lead to a relatively large *s*. With increasing concentration, intermolecular interactions gradually increase as well. Thus, *s* generally decreases with increasing γ-PGA concentration. Furthermore, we can see that *s* is in direct proportion to the concentration of γ-PGA (Fig. 2(e)). This is reasonable since the experimental concentration range is not high. The equation $s={s}_{0}(1-{k}_{s}c)$ can be used here, where s_0_ represents the sedimentation coefficient in infinite dilute solution and *k*_s_ is the concentration coefficient.

### Morphology of γ-PGA-H and γ-PGA-Na

**Figure 3:**
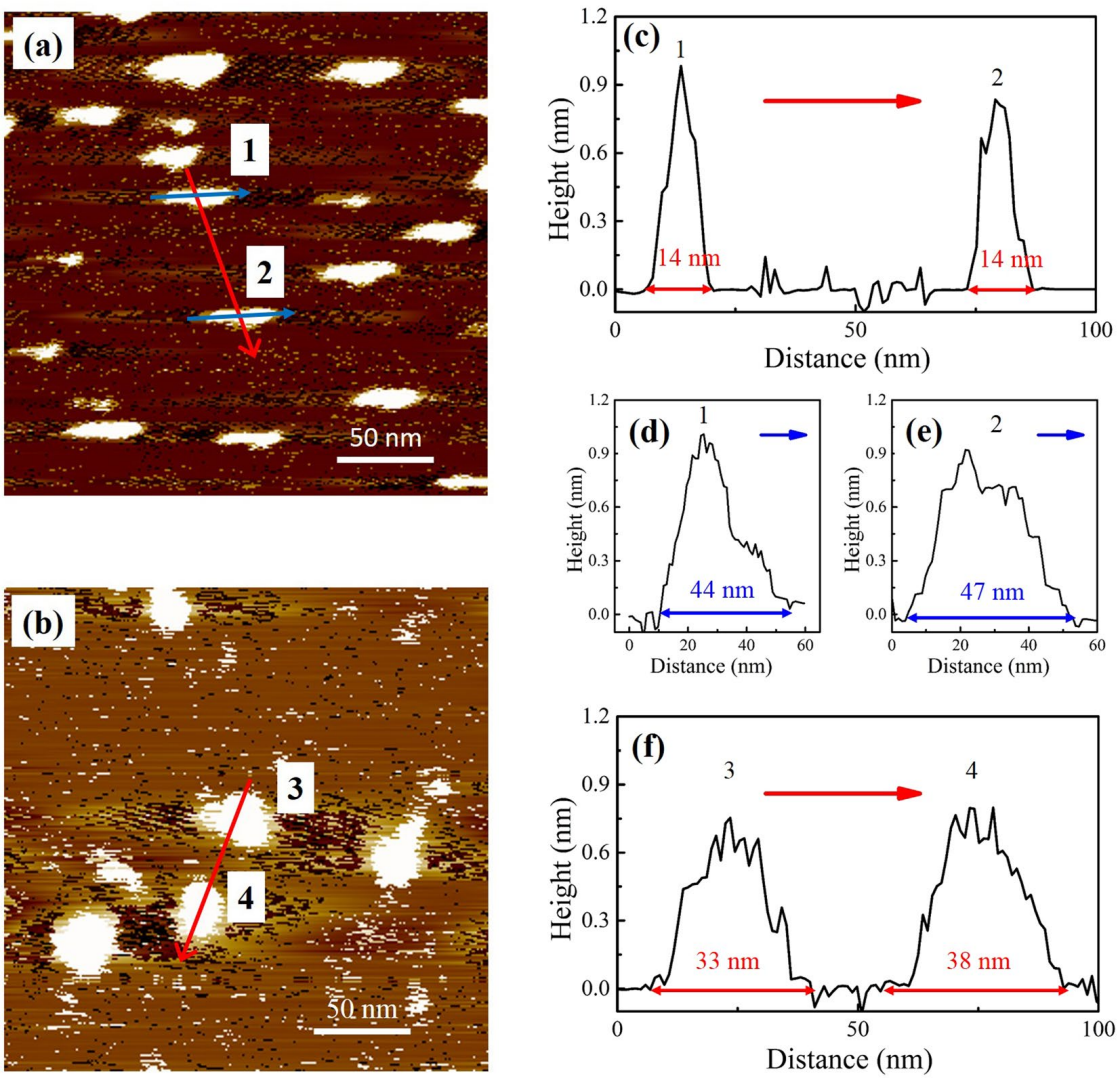
AFM images of the acid form γ-PGA-H (**a**) and sodium form γ-PGA-Na (**b**). Cross-sectional images of particles 1 and 2 ((**c**), (**d**), (**e**)). Cross-sectional images of particles 3 and 4 (**f**).

The morphology of the acid form γ-PGA-H and sodium form γ-PGA-Na were observed using AFM (Fig. 3). The structure of γ-PGA-H (Fig. 3(a)) was clearly distinct from that of γ-PGA-Na (Fig. 3(b)). The γ-PGA structures presented in the AFM images should represent the shape of γ-PGA molecular and the size was considered as the rotating radius (*R*_R_), since the γ-PGA-H and γ-PGA-Na solutions used in AFM were more dilute (0.01 g/L) than in the AUC measurements.

γ-PGA-H exhibited rod-like shapes and coexisted as both small and large particles (Fig. 3(a and b)). For example, the widths of two typical particles were both 14 nm, while their lengths were 44 nm and 47 nm (Fig. 3(a)). When a sodium ion replaced a hydrogen ion in the γ-PGA structure, the rod-like molecular structure changed to a sphere-like shape (Fig. 3(b)). The diameters of two typical γ-PGA-Na spheres were approximately 33 nm and 38 nm. The heights in the cross-sectional images (Fig. 3(c–f)) show that the particles collapsed upon drying in air and were present as flat structures on the mica surface. When compared to the γ-PGA-H structure, the γ-PGA-Na structure appeared to have a larger volume.

### Conformation of γ-PGA at different pH values

**Figure 4:**
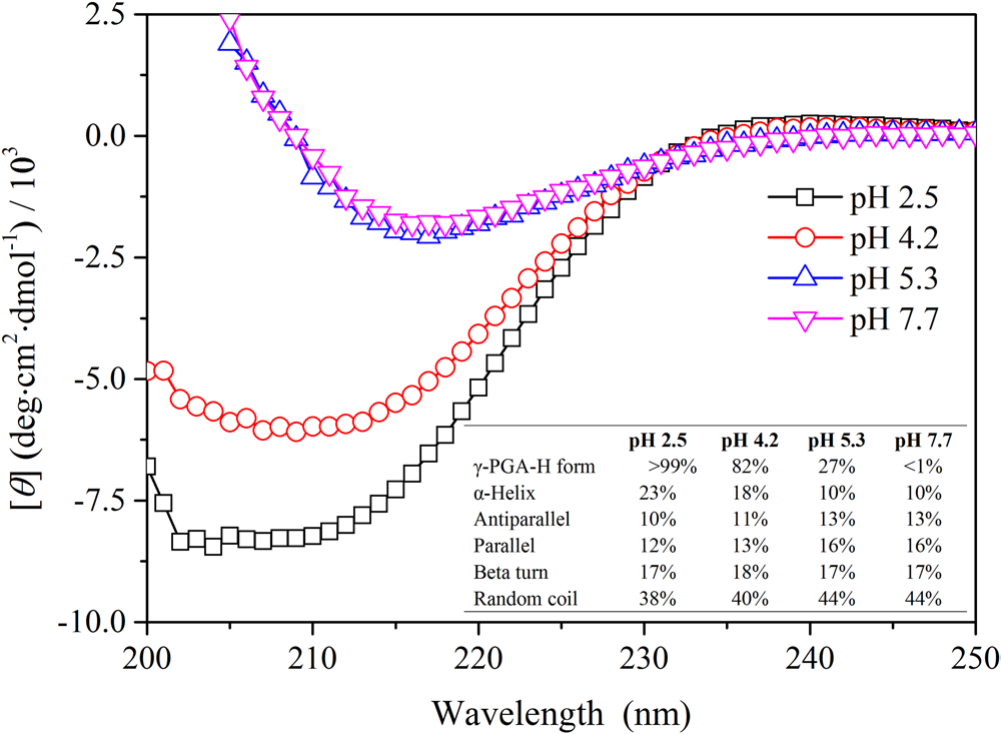
Effect of pH on γ-PGA CD spectroscopy; the inset represents the secondary structure results fitted by the CD spectra deconvolution software CDNN 2.1.

CD spectroscopy and LLS were used to further explore the conformations of γ-PGA at different pH values. Here, the effects of pH on γ-PGA was investigated using CD spectroscopy. The secondary structure results were determined using the CD spectra deconvolution software CDNN 2.1 (Fig. 4). The secondary structure of the γ-PGA-H form (pH 2.5) consisted of 23% α-helix, 10% antiparallel, 12% parallel, 17% beta turn, and 38% random coil. With the decrease in γ-PGA-H percentage (i.e.: an increase of COO^−^ groups in the γ-PGA side chains) there was a substantial decrease in the α-helix from 23% to 10% and a considerable increase in the antiparallel, parallel, and random coil from 10% to 13%, 12% to 16%, and 38% to 44%, respectively. These results indicate that the structure of the γ-PGA changes after ionization of the COOH groups in the γ-PGA side chains.

**Figure 5:**
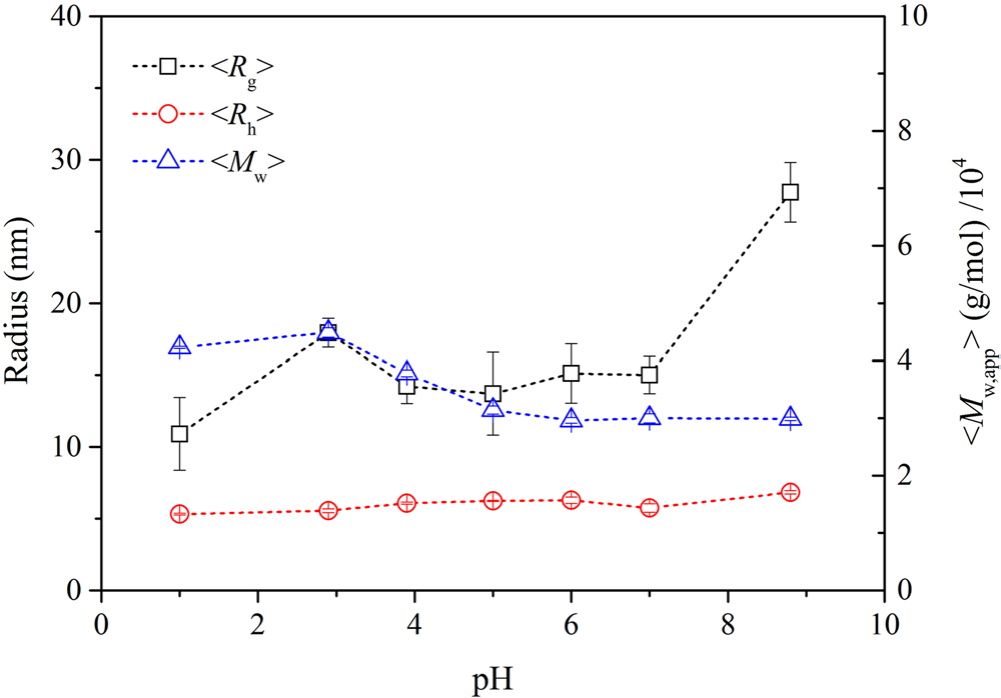
Effect of pH on <*R*_*g*_>, <*R*_*h*_>, and the apparent weight average molar mass, <*M*_w,app_>, of γ-PGA (3 g/L) as measured by LLS.

Sizes of γ-PGA, including <*R*_*g*_> and <*R*_*h*_>, and the apparent weight average molar mass <*M*_w,app_> at various pH values were determined using LLS measurements (Fig. 5). The <*R*_*g*_> of γ-PGA initially increased from pH 1.0 to 2.9, then steadied from pH 2.9 to 7.0, and then further increased at pH 8.8. These results were consistent with the AFM image data, thereby indicating that the γ-PGA-Na structure appeared to have a larger volume. Compared to the solely geometrically defined <*R*_g_>, <*R*_h_> is differently defined and independent. The much smaller value of <*R*_*h*_>, when compared to that of <*R*_*g*_>, suggests that the γ-PGA structure was deep drained by the water, so that the γ-PGA molecules collapsed and were present as flat structures on the mica surface in AFM images. The values of *ρ* (*ρ* = <*R*_g_>/<*R*_*h*_>) were 2.1, 3.2, 2.3, 2.2, 2.4, 2.6 and 4.1 at pH 1.0, 2.9, 3.9, 5.0, 6.0, 7.0 and 8.8 respectively. The <*R*_*h*_> of γ-PGA only slightly increased from 5.31 nm to 6.83 nm with increasing pH, likely arising from the structural change from α-helix to random coil and increased ionic hydration. The ionic hydration γ-PGA may increase with the increasing degree of ionization and attraction of cationic counter ions at higher pH values. However, the <*M*_w,app_> of γ-PGA reduced gradually from 4.2 × 10^4^ g/mol to 3.0 × 10^4^ g/mol as the pH increased.

### FTIR spectra of γ-PGA

**Figure 6:**
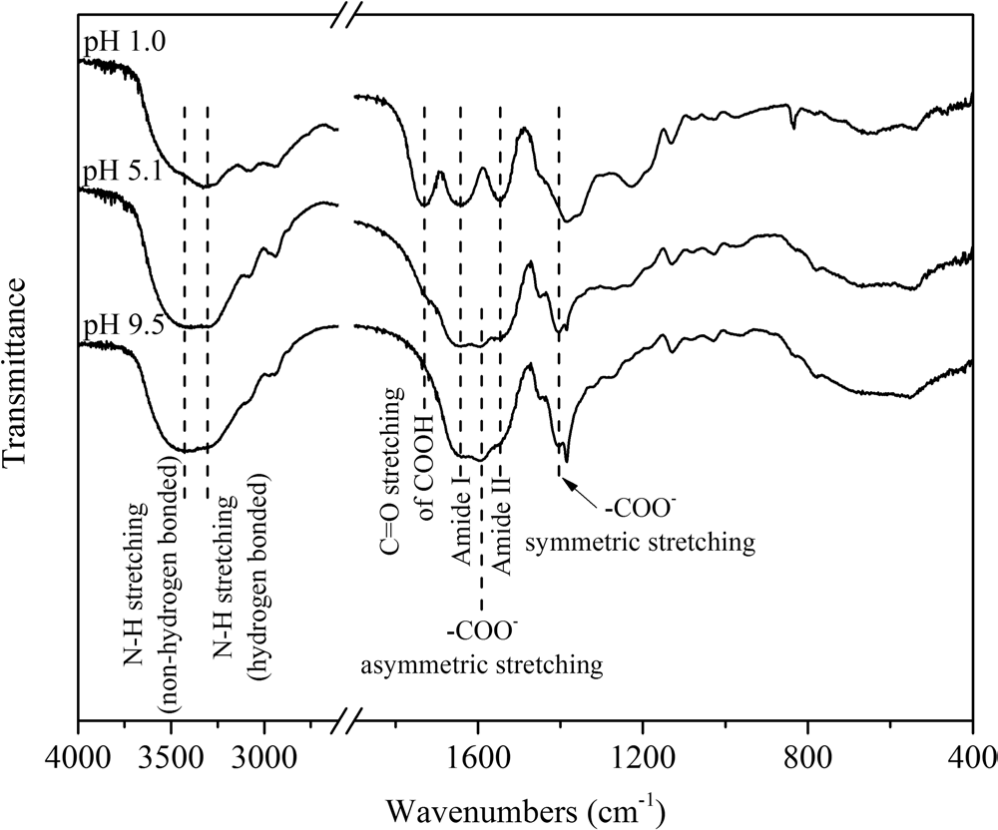
FTIR spectra of lyophilized γ-PGA at pH 1.0, 5.1, and 9.5.

At lower pH, it has been suggested that intramolecular hydrogen bonds are the dominant molecular force associated with γ-PGA^21^. FTIR spectra were used to confirm the existence of the hydrogen bonds in γ-PGA at pH 1.0 (99.9% γ-PGA-H form), 5.1 (36.5% γ-PGA-H form), and 9.5 (<0.1% γ-PGA-H form). In the γ-PGA-H form, the broad peak at 3310 cm^−1^ (Fig. 6) corresponded to hydrogen-bonded N–H stretching while the shoulder, which appears at around 3430 cm^−1^, was attributable to non-hydrogen-bonded N–H^22^. With an increase in pH, the shoulder intensity increased due to the continuous dissociation of hydrogen bonds.

A distinct peak around 1730 cm^−1^ at pH 1.0, which gradually disappeared at pH 5.1 and 9.5, could be assigned to the C=O stretching in COOH. With the deprotonation of COOH, the C=O in the zwitterionic form COO^−^ shifted to about 1590 cm^−1^ (asymmetric stretching) and 1410 cm^−1^ (symmetric stretching)^23^. The intensity at 1590 cm^−1^ even overlapped the amide I and amide II bands at pH 5.1 and 9.5. The amide I band around 1643 cm^−1^ at pH 1.0 primarily originated from the C=O stretching vibration in the γ-PGA-H amide group because the amide I band represented 80% C=O stretching vibration of the amide group coupled to in-plane N–H bending and C–N stretching modes^24^. A small shift to 1653 cm^−1^ in the amide I band at pH 5.1 and 9.5 may arise from the dissociation of hydrogen bonds with the N–H.

## Discussion

Conventional viscosity studies of γ-PGA, and other SMPs, have focused on its relationship with concentration, molecular weight, and temperature^25–28^. However, the observation that the addition of acid to the medium effectively reduces the viscosity of the culture broth has not been reasonably explained; however, it has allowed for development of an efficient method to separate and recover γ-PGA from highly viscous culture broth, which minimizes purification and recovery costs for large-scale industrial appliations^29^. Therefore, in our study we focused on the relationship between viscosity and pH. We found that the observed results agree with previously published research^29^. At a defined concentration, molecular weight, and temperature, conformational changes and molecular interactions may be the primary cause for the significant increase in viscosity as pH increases.

The sizes determined by AFM in the dried state are different from those radii measured by LLS in the aqueous solution, presumably due to the different definition and sample preparation. AFM clearly demonstrates the distinct structures of the γ-PGA-H and γ-PGA-Na forms, thereby confirming conformational changes of γ-PGA. The decrease of α-helix and increase of random coil in the γ-PGA secondary structure, as analyzed by CD, are considered to be the main conformation responsible for the shape variation at different pH values. A similar helix to random coil structural change associated with pH variation has been substantiated for poly(glutamic acid) using CD studies^30,31^. The conformation of the poly(glutamic acid) was reported to change from random coil to α-helix as the solution pH decreased from 7.2 to 3.0^30^. Another study of γ-PGA, which was purified from *B. licheniformis*, used attenuated total reflectance FTIR (ATR-FTIR) to show that the exopolymer (0.1% w/v) is protonated and exhibits a helical conformation at a low pH, whereas a β-sheet-based conformation predominates at higher pH^13^.

**Figure 7:**
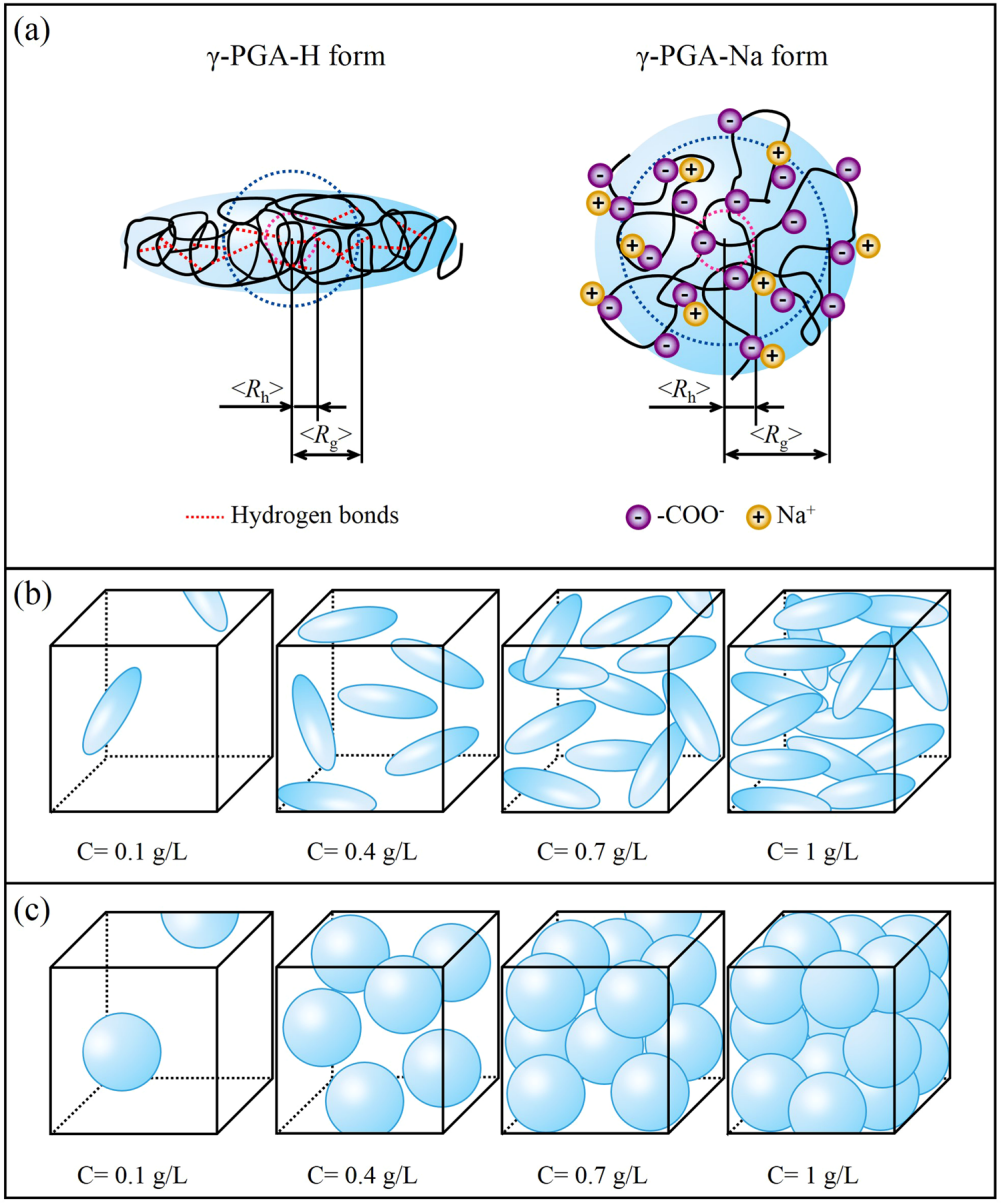
Schematic structural illustrations of the acid form γ-PGA-H and sodium form γ-PGA-Na (**a**), concentrations of γ-PGA-H (**b**) and γ-PGA-Na (**c**) in aqueous solutions at 0.1, 0.4, 0.7, and 1.0 g/L, used in AUC measurements. The side length of the cube is 100 nm in (**b**) and (**c**).

Conformations of the γ-PGA-H acid form and γ-PGA-Na sodium form are schematically illustrated in Fig. 7(a). The γ-PGA-H structure is stabilized by intramolecular hydrogen bonds. The un-ionized state of poly (γ-D-glutamic acid) was investigated using a combination of molecular dynamics and quantum mechanical calculations^21^. Theoretical models indicate that a left-handed helix with a 19-membered ring and hydrogen bonds set between the CO of the amide group *i* and the NH of amide group *i* + 3 is the most stable conformation. Weak intramolecular interactions between the side carboxylic oxygen and the NH of the backbone amide group are assumed to be responsible for the relatively high stability of the left-handed helix. More interestingly, hydrogen bonds in poly(γ-D-glutamic acid) would be three-centered after incubation at 65 °C for 48 h^32^. The α-helical conformation is metastable and transforms spontaneously into insoluble β-fibrils aggregates with the characteristic infrared trait. This has been attributed to the network of three-centered hydrogen bonds coupled to the side chain’s carboxyl and the main chain’s −NH groups^32–34^. The strong intramolecular hydrogen bonds between the CO and the NH of the amide group were confirmed by FTIR. However, the evidence of presumed hydrogen bonds between carboxylate groups of the side chains and NH of the amide group were not evident within the FTIR spectra. It is likely that these hydrogen bonds are weak intramolecular interactions.

The random coil structure adopted by the γ-PGA-Na molecule may be attributed to two factors. Firstly, the deprotonated carboxylate groups destabilize and are therefore not available to maintain the hydrogen bonds between the side chains and the backbone. Secondly, each monomer of the γ-PGA molecule has one carboxylate group and this negatively charged carboxylate group has a strong electrostatic repulsion, which is resistant to the intramolecular interaction of hydrogen bonds between the CO and NH of the amide group.

Changes in molecular interactions of γ-PGA solutions at different pH were also confirmed by AUC measurements. Figure 7(b) and (c) illustrate the concentrations of γ-PGA-H and γ-PGA-Na in solutions at 0.1, 0.4, 0.7, and 1.0 g/L, which were used in AUC measurements. In a dilute solution (0.1 g/L), the molecules are separated from each other and behave independently. The polymer chain interacts primarily with the solvent molecules; therefore, the intermolecular interactions of γ-PGA in dilute solutions can be neglected. There were no significant differences in the sedimentation coefficient distributions for 0.1 g/L γ-PGA at pH 3.8, 5.9, and 8.9 (Fig. 2(a)) despite the structures of γ-PGA being distinct at these three pH values. These results imply that the sedimentation coefficient distributions of γ-PGA are dependent on the intermolecular interaction, rather than the intramolecular interaction.

In contrast, as the γ-PGA concentration increased, the variation of structures associated with pH affected the peaks and distribution of the sedimentation coefficient (Fig. 2(c–e)). According to the definition of sedimentation coefficient, *s*, a larger *s* represents a faster sedimentation velocity. The peaks and distribution of the sedimentation coefficient were shifted to slower sedimentation velocities as the pH increased with increasing concentrations from 0.1 to 1.0 g/L. These results indicate that the intermolecular interactions increase as the pH increases. Furthermore, the narrowed sedimentation coefficient distributions at higher pH values and increased concentrations suggest that the intermolecular interactions make the denser γ-PGA more homogeneous at higher concentrations.

Generally, the most probable intermolecular interactions in high concentration solutions of γ-PGA are electrostatic repulsion and chain entanglement. As expected, the negative charge derived from deprotonation of carboxylate groups increases with increasing pH, resulting in stronger intermolecular repulsion at a higher pH. The second virial coefficient, *A*_2_, of γ-PGA at pH 7.0 (phosphate buffer, 0.13 M ionic strength) was previously reported as a positive value (*A*_2_ = 1.44 × 10^−6^ mol·L/g^2^)^35^, which reflects repulsion between γ-PGA molecules at pH 7.0. Chain entanglement^18^ is considerable when the γ-PGA molecules exist in a high concentration solution. Furthermore, the random coil structure of γ-PGA-Na likely promotes chain overlap and entanglement. Additionally, the stable conformation of γ-PGA at higher concentrations is probably due to the balance of intramolecular and intermolecular electrostatic repulsion and intermolecular chain entanglement.

Compared to the extracellular polymeric substances bounded to the microorganisms, the excreted SMPs are well dissolved in aqueous solutions and have much larger values of *ρ* ^36,37^. The *ρ* value of bound extracellular polymeric substances of *Bacillus megaterium* TF10 with a negative *A*_2_ (*A*_2_ = −2.9 × 10^−8^ mol·L/g^2^) at pH 7.0 and 0.1 M ionic strength was about 1.2^36^ while the *ρ* value of its SMP was about 2.6 with a positive *A*_2_ (*A*_2_ = 1.7 × 10^−6^ mol·L/g^2^, similar to the positive *A*_2_ of γ-PGA) at pH 7.0 and 0.75 M ionic strength^37^. The large values of *ρ* in aqueous solutions might confirmed that the γ-PGA is a well dissolved SMP.

## Methods

### γ-PGA purification

A γ-PGA solution (5% w/w), produced from *B. licheniformis* ATCC 9945 A, was supplied by Guangdong Demay biotechnology Co., China. The γ-PGA solution was concentrated and then washed repeatedly with deionized water by using a cross flow ultrafilter equipped with a Millipore Pellicon 2 Cassette (10 K Da molecular-weight cutoff polyethersulfone membrane) to remove ions and small molecules. The resulting γ-PGA solution was then filtered through a 0.22 μm hydrophilic PTFE filter and subsequently lyophilized. A portion of the lyophilized γ-PGA was further dissolved in distilled water and adjusted with excess HNO_3_ or NaOH, and then dialyzed and lyophilized to obtain the acid form (γ-PGA-H) and sodium salt form (γ-PGA-Na) of γ-PGA for AFM images.

### γ-PGA characterization

The purity of γ-PGA was determined by amino acid analysis using thin layer chromatography (TLC) after hydrolysis, according to Yokoi *et al*.^38^. Sole detection of glutamic acid in the TLC analysis confirmed the purity of γ-PGA (data not shown). The hydrolyzed γ-PGA product was further used to determine the glutamic acid content by colorimetric analysis, with glutamic acid as the standard. The glutamic acid content was determined as 7.02 ± 0.08 mmol/g. Metal ions were analyzed on an inductively coupled plasma mass spectrometer (ICP-MS, 7700X, Agilent Inc., USA). The most abundant metal ions were Na, Mg, K, and Ca, which were determined to be 29.69 ± 0.33 mg/g, 0.46 ± 0.01 mg/g, 0.74 ± 0.01 mg/g and 0.67 ± 0.03 mg/g, respectively. Based on the glutamic acid and metal ion results, the γ-PGA was determined to be primarily composed of the acid form (γ-PGA-H; 81.62%), with the remainder existing in the sodium salt form (γ-PGA-Na; 18.38%). The purity of γ-PGA was determined as 94 wt %. The pK_a_ value of γ-PGA was determined to be 4.86, based on the previously reported acid-base titration method^39^ using an automatic titrator (TIM865, Hach Co., USA).

### Viscosity assay

The viscosity of γ-PGA at different pH values was analyzed on a BGD 155 Digital Viscometer (Biuged Laboratory Instruments, Guangzhou, China) at 20 °C. The concentration of γ-PGA used for viscosity analysis was 5% w/v and the pH was adjusted with HNO_3_ or NaOH, with NaNO_3_ added to control the ionic strength (I = 0.2 M).

### Analytical ultracentrifugation (AUC) measurements

The analytical ultracentrifuge (AUC) technique was used to characterize the sedimentation coefficient based on sedimentation velocity (SV) analysis^40,41^. SV experiments were performed on a Proteomelab XL-A analytical ultracentrifuge (Beckman Coulter Instruments) coupled with a UV-vis absorption optics detector to monitor the time-dependent radial concentration of γ-PGA from 190 to 800 nm. Three cells equipped with a two-sector, charcoal-filled Epon centerpiece, quartz windows and a counterbalance were loaded into an An-60 Ti 4-hole rotor. γ-PGA solution (400 μL) and solvent (410 μL) were added as the sample and reference, respectively. Na_2_HPO_4_ and Na_2_HPO_4_ were used to adjust the pH of γ-PGA solution because HNO_3_ and NaOH disrupt the signal from 190 to 260 nm. The ionic strength of the γ-PGA solution was kept at 0.2 M by addition of NaCl so that the net charges were completely screened. γ-PGA solution was thermostated at 20 °C and 0 rpm for at least 2 h before the angular velocity increased to the final rotational speed of 58,000 rpm. Approximately 200 scans of data for every sample were collected at a time interval of 3 min and were analyzed using the SEDFIT software program^42,43^. The Z-scores as well as the rmsd values of the fits in relation to that expected from the noise in the optical system are provided in Supplementary Information (Table S1). The Lamm equation describes the sedimentation and diffusion processes of molecules during centrifugation:

1 $$\frac{\partial c}{\partial t}=\frac{1}{r}\frac{\partial }{\partial r}[r\cdot D\frac{\partial c}{\partial r}-{\omega }^{2}{r}^{2}sc]$$

where *c*, *t*, *r*, *D*, *ω* and *s* are the concentration of the solute, sedimentation time, radial distance from the axis of rotation, diffusion coefficient, angular velocity and sedimentation coefficient, respectively. The sedimentation coefficient is defined as *s* = *u*/*ω* ^2^ *r* where *u* is the sedimentation velocity of the solute.

Partial specific volume (*v*), an important and essential parameter, is defined as the change in volume when a measured amount of solute is added to the solution. It can be calculated from the following equation:

2 $$\nu =(1-\frac{{\rm{\Delta }}\rho }{{\rm{\Delta }}c})/{\rho }_{0}$$

where *ρ*_0_ is the density of solvent, *ρ* is the density of solution, and *c* is the concentration of the solute. The density values obtained from the solutions at different concentrations were plotted against the concentration values, with $\frac{\Delta \rho }{\Delta c}$ as the slope. The partial specific volume of the γ-PGA solution was measured using a DMA4500 densitometer (Anton Paar) and was determined to be 0.656, 0.715, and 0.730 mL/g at pH 3.8, 5.9, and 8.9, respectively.

Using the value of *s* and weight-average frictional ratio *f/f*_0_ fitted from *c*(*s*) model and the parameters of partial-specific volume, density and viscosity of the solution, diffusion coefficient *D* can be calculated with the combination of Svedberg equation and Stokes-Einstein equation. Then, once the *D* is obtained, the hydrodynamic radius can be calculated from Stokes-Einstein equation.

### Atomic force microscope (AFM) imaging

AFM (Park Systems, XE-100) images were obtained to characterize the morphology of the γ-PGA-H and γ-PGA-Na forms. Approximately 2 drops of the γ-PGA-H or γ-PGA-Na solution (0.01 g/L) were applied to a clean mica sheet and spin-coated (3000 rev/min) for 60 s in air. After the mica sheet dried, AFM images were acquired using a Park Systems microscope (XE-100) operating in non-contact mode at scanning rates of 0.3 or 0.5 with an image resolution of 256 by 256. The Silicon cantilever (PPP-NCHR, Nanosensors) used in the AFM was carried out with a spring constant of 42 N/m and resonance frequency of 330 kHz.

### Circular dichroism (CD) measurements

CD measurements of γ-PGA solutions (0.1 g/L, in 0.1 mol/L NaF ionic medium) were performed on a Chirascan spectrometer (Applied Photophysics Co., UK) using a quartz cell of 1 cm path length. CD spectra were collected from 190 to 260 nm. Again, H_3_PO_4_ and Na_2_HPO_4_ were used to adjust the pH, as HNO_3_ and NaOH would disrupt the signal of γ-PGA from 190 to 260 nm. The mean residue molar ellipticities were calculated according to the following equation^44^:

3 $$[\theta ]={[\theta ]}_{{\rm{obs}}}\frac{{\rm{MRW}}}{10{lc}}$$

where [*θ*]_obs_ is the observed ellipticity measured in degrees, MRW is the mean residue weight, *l* is the path length of the cell in centimeters, and *c* is the molar concentration of γ-PGA in g/L. CD spectra deconvolution software CDNN 2.1 (courtesy of Gerald Böhm, Martin-Luther-Universität Halle-Wittenberg, Germany) was used to analyze the secondary structure of γ-PGA.

### Laser light scattering (LLS) measurements

LLS measurements were conducted according to our previous studies^36,39^ on an ALV/DLS/SLS-5022F spectrometer equipped with a multi-τ digital time correlator (ALV5000) and a cylindrical 22 mW UNIPHASE He-Ne laser (*λ*_0_ = 632.8 nm) as the light source. The γ-PGA solutions were filtered using a 0.45 μm hydrophilic PTFE (Millex-LCR, Millipore Inc., USA) into a dust-free vial and tested at 20.0 ± 0.1 °C. The pH of the γ-PGA solution was adjusted with HNO_3_ or NaOH, with NaNO_3_ added to control the ionic strength (I = 0.2 M).

In static light scattering (SLS), the excess Rayleigh ratio, *R*_ex_, is related to the weight-averaged molecular weight (*M*_w_) and the *z*-average root-mean-square radius of gyration <*R*_g_^2^>_z_^1/2^ (or simply <*R*_*g*_>) as described by^39^:

4 $$\frac{KC}{{R}_{ex}}\approx \frac{1}{{M}_{w,app}}(1+\frac{1}{3}{\langle {{R}_{g}}^{2}\rangle }_{z}{q}^{2})+2{A}_{2}C$$

where *K* = 4*π* ^2^ *n* ^2^(*dn*/*dC*)^2^/(*N*_A_ *λ*_0_^4^) with *n*, *dn*/*dC*, *N*_A_, and *λ*_0_ representing the solvent refractive index, the specific refractive index increment, Avogadro’s number, and the wavelength of light in vacuum, respectively. *q* = (4*πn*/*λ*_0_)sin(*θ*/2) with *θ* being the scattering angle. The value of *dn*/*dC* (specific refractive index increment) was determined with a precise differential refractometer at 20.0 °C and 633 nm. In SLS measurements, the intensity of scattered light at each angle was measured three times, indicating a variation of less than 5%. The relative errors of <*R*_*g*_> and *M*_w,app_ were ±8% and ±5%.

In dynamic light scattering (DLS), the precisely measured intensity-intensity time correlation function *G* ^(2)^(*q*, *τ*) was measured in the self-beating mode and the Laplace inversion of *G* ^(2)^(*q*, *τ*) led to a line width distribution *G*(*Γ*). For a diffusive relaxation, *Γ* is directly related to the translational diffusion coefficient, *D*, which can be further converted to the hydrodynamic radius, <*R*_*h*_>, by using the Stokes-Einstein equation. The cumulant expansion analysis was used in the DLS data and the detailed theoretical analysis was provided in our previous study^36^. The *G* ^(2)^(*q*, *τ*) at each pH value was measured four times and the standard deviation (StdDev) for each fitting was listed in Supplementary Information (Table S2).

### FTIR spectroscopy analysis

FTIR spectra of the samples were recorded on an FTIR-7600 instrument (Lambda Scientific Pty Ltd., USA). The pH of the γ-PGA samples (10 mg/mL in deionized water) was adjusted to approximately 1.3, 7.0 and 10.0 using 0.2 M HCl or NaOH. The prepared samples were then lyophilized and directly mixed into FTIR grade KBr powder for testing.

## Electronic supplementary material

Supplementary Information

## References

[1] Laspidou CS, Rittmann BE (2002). A unified theory for extracellular polymeric substances, soluble microbial products, and active and inert biomass. Water Res..

[2] More TT, Yadav JSS, Yan S, Tyagi RD, Surampalli RY (2014). Extracellular polymeric substances of bacteria and their potential environmental applications. J. Environ. Manage..

[3] Tourney J, Ngwenya BT (2014). The role of bacterial extracellular polymeric substances in geomicrobiology. Chem. Geol..

[4] Bhatnagar A, Sillanpaa M (2017). Removal of natural organic matter (NOM) and its constituents from water by adsorption - a review. Chemosphere.

[5] Ni BJ, Yu HQ (2012). Microbial products of activated sludge in biological wastewater treatment systems: a critical review. Crit. Rev. Environ. Sci. Technol..

[6] Yu H (2015). Relationship between soluble microbial products (SMP) and effluent organic matter (EfOM): characterized by fluorescence excitation emission matrix coupled with parallel factor analysis. Chemosphere.

[7] Bajaj I, Singhal R (2011). Poly (glutamic acid) - an emerging biopolymer of commercial interest. Bioresour. Technol..

[8] Matilainen A, Vepsalainen M, Sillanpaa M (2010). Natural organic matter removal by coagulation during drinking water treatment: a review. Adv. Colloid Interface Sci..

[9] Wang Z, Zhang L, Zhao J, Xing B (2016). Environmental processes and toxicity of metallic nanoparticles in aquatic systems as affected by natural organic matter. Environ.-Sci. Nano.

[10] Kunacheva C, Stuckey DC (2014). Analytical methods for soluble microbial products (SMP) and extracellular polymers (ECP) in wastewater treatment systems: a review. Water Res..

[11] Ni BJ, Rittmann BE, Yu HQ (2011). Soluble microbial products and their implications in mixed culture biotechnology. Trends Biotechnol..

[12] Causse B (2016). Xanthan exopolysaccharide: Cu^2+^ complexes affected from the pH-dependent conformational state; implications for environmentally relevant biopolymers. Environ. Sci. Technol..

[13] He LM, Neu MP, Vanderberg LA (2000). *Bacillus lichenformis* gamma-glutamyl exopolymer: physicochemical characterization and U(VI) interaction. Environ. Sci. Technol..

[14] Buescher JM, Margaritis A (2007). Microbial biosynthesis of polyglutamic acid biopolymer and applications in the biopharmaceutical, biomedical and food industries. Crit. Rev. Biotechnol..

[15] Ogunleye A (2015). Poly-gamma-glutamic acid: production, properties and applications. Microbiology-(UK).

[16] Candela T, Fouet A (2006). Poly-gamma-glutamate in bacteria. Mol. Microbiol..

[17] Shih IL, Van YT (2001). The production of poly-(gamma-glutamic acid) from microorganisms and its various applications. Bioresour. Technol..

[18] Teraoka, I. Models of polymer chains in *Polymer solutions: an introduction to physical propertie*s 64–65 (2002).

[19] Tang B (2015). Highly efficient rice straw utilization for poly-(gamma-glutamic acid) production by *Bacillus subtilis* NX-2. Bioresour. Technol..

[20] Wang XY, Ye XD, Zhang GZ (2015). Investigation of pH-induced conformational change and hydration of poly(methacrylic acid) by analytical ultracentrifugation. Soft Matter.

[21] Zanuy D, Aleman C, Munoz-Guerra S (1998). On the helical conformation of un-ionized poly(gamma-D-glutamic acid). Int. J. Biol. Macromol..

[22] Appoh FE, Thomas DS, Kraatz HB (2005). Glutamic acid dendrimers attached to a central ferrocene core: synthesis and properties. Macromolecules.

[23] Garcia-Alvarez M (2005). Comb-like ionic complexes of cationic surfactants with bacterial poly(gamma-glutamic acid) of racemic composition. Macromol. Biosci..

[24] Stuart, B. H. Biological applications in *Infrared spectroscopy: fundamentals and application*s 142 (2004).

[25] Irurzun I (2001). Mark-Houwink parameters of biosynthetic poly(gamma-glutamic acid) in aqueous solution. Macromol. Chem. Phys..

[26] Richard A, Margaritis A (2001). Poly(glutamic acid) for biomedical applications. Crit. Rev. Biotechnol..

[27] Wu Q, Xu H, Liang J, Yao J (2010). Contribution of glycerol on production of poly(gamma-glutamic acid) in *Bacillus subtilis* NX-2. Appl. Biochem. Biotechnol..

[28] Zhu F (2014). A novel approach for poly-gamma-glutamic acid production using xylose and corncob fibres hydrolysate in *Bacillus subtillis* HB-1. J. Chem. Technol. Biotechnol..

[29] Do JH, Chang HN, Lee SY (2001). Efficient recovery of gamma-poly (glutamic acid) from highly viscous culture broth. Biotechnol. Bioeng..

[30] Agresti C, Tu Z, Ng C, Yang Y, Liang JF (2008). Specific interactions between diphenhydramine and alpha-helical poly(glutamic acid) - a new ion-pairing complex for taste masking and pH-controlled diphenhydramine release. Eur. J. Pharm. Biopharm..

[31] Gooding EA (2013). pH-dependent helix folding dynamics of poly-glutamic acid. Chem. Phys..

[32] Fulara A, Dzwolak W (2010). Bifurcated hydrogen bonds stabilize fibrils of poly(L-glutamic) acid. J. Phys. Chem. B.

[33] Hernik-Magon A (2016). Beware of cocktails: chain-length bidispersity triggers explosive self-assembly of poly-L-Glutamic acid beta(2)-fibrils. Biomacromolecules.

[34] Yamaoki Y (2012). An FT-IR study on packing defects in mixed beta-aggregates of poly(L-glutamic acid) and poly(D-glutamic acid): a high-pressure rescue from a kinetic trap. J. Phys. Chem. B.

[35] de Cesaro A, da Silva SB, da Silva VZ, Zachia Ayub MA (2014). Physico-chemical and rheological characterization of poly-gamma-glutamic acid produced by a new strain of *Bacillus subtilis*. Eur. Polym. J..

[36] Wang LL (2012). Spatial configuration of extracellular polymeric substances of *Bacillus megaterium* TF10 in aqueous solution. Water Res..

[37] Wang LL, Wang LF, Ye XD, Yu HQ (2013). Hydration interactions and stability of soluble microbial products in aqueous solutions. Water Res..

[38] Yokoi H, Natsuda O, Hirose J, Hayashi S, Takasaki Y (1995). Characteristics of a biopolymer flocculant produced by *Bacillus* Sp. Py-90. J. Ferment. Bioeng..

[39] Wang LL (2012). pH dependence of structure and surface properties of microbial EPS. Environ. Sci. Technol..

[40] Laue TM, Stafford WF (1999). Modern applications of analytical ultracentrifugation. Annu. Rev. Biophys. Biomolec. Struct..

[41] Mächtle, W. & Börger, L. Sedimentation velocity In *Analytical ultracentrifugation of polymers and nanoparticle*s 62–64 (2006).

[42] Lebowitz J, Lewis MS, Schuck P (2002). Modern analytical ultracentrifugation in protein science: a tutorial review. Protein Sci..

[43] Schuck P (2000). Size-distribution analysis of macromolecules by sedimentation velocity ultracentrifugation and Lamm equation modeling. Biophys. J..

[44] Joyce J (2006). 2006. Immunogenicity and protective efficacy of *Bacillus anthracis* poly-gamma-D-glutamic acid capsule covalently coupled to a protein carrier using a novel triazine-based conjugation strategy. J. Biol. Chem..

