# Supplementary material for: Conformations and molecular interactions of poly-γ-glutamic acid as a soluble microbial product in aqueous solutions
Source: Sci Rep. 2017 Oct 6;7:12787. doi: 10.1038/s41598-017-13152-2 (PMC5630630; doi:10.1038/s41598-017-13152-2)
Supplement: Supplementary file 1 — Supplementary Information [file 41598_2017_13152_MOESM1_ESM.doc]

Supplementary information

**Conformations and** **molecular interactions of** **poly-γ-glutamic acid as a soluble microbial product in aqueous solutions**

Ling-Ling Wang^1^, Jian-Tao Chen^2^, Long-Fei Wang^3^, Sha Wu^4^, Guang-zhao Zhang^2^, Han-Qing Yu^3^, Xiao-dong Ye^4^, Qing-Shan Shi^*,1^

^1^State Key Laboratory of Applied Microbiology Southern China, Guangdong Provincial Key Laboratory of Microbial Culture Collection and Application, Guangdong Open Laboratory of Applied Microbiology, Guangdong Institute of Microbiology, Guangzhou, 510070, China

^2^Faculty of Materials Science and Engineering, South China University of Technology, Guangzhou, 510640, China

^3^Department of Chemistry, ^4^Hefei National Laboratory for Physical Sciences at the Microscale, Department of Chemical Physics, University of Science and Technology of China, Hefei, 230026, China

*** Corresponding author:**

Prof. Qing-Shan Shi, Fax: +86-20-87137652; E-mail: shiqingshan@hotmail.com

**Methods**

| γ-PGA concentration (mg/ml) | Runs-Z test score | r.m.s.d. |
| --- | --- | --- |
| pH = 3.8 | | |
| 0.1 | 3.6 | 0.009 |
| 0.4 | 11.6 | 0.008 |
| 0.7 | 25.8 | 0.005 |
| 1.0 | 45.4 | 0.007 |
| pH = 5.9 | | |
| 0.1 | 3.6 | 0.009 |
| 0.4 | 17.3 | 0.008 |
| 0.7 | 28.0 | 0.005 |
| 1.0 | 61.9 | 0.007 |
| pH = 8.9 | | |
| 0.1 | 2.2 | 0.009 |
| 0.4 | 15.1 | 0.008 |
| 0.7 | 24.2 | 0.005 |
| 1.0 | 51.6 | 0.008 |

Table S1: Z-scores and rmsd values of the fits in relation to that expected from the noise in the AUC optical system.

| pH | StdDev 1 | StdDev 2 | StdDev 3 | StdDev 4 |
| --- | --- | --- | --- | --- |
| 1.0 | 0.0119 | 0.0109 | 0.0066 | 0.0062 |
| 2.9 | 0.0118 | 0.0096 | 0.0077 | 0.0096 |
| 3.9 | 0.0128 | 0.0010 | 0.0069 | 0.0078 |
| 5.0 | 0.0137 | 0.0104 | 0.0095 | 0.0082 |
| 6.0 | 0.0146 | 0.0111 | 0.0093 | 0.0090 |
| 7.0 | 0.0140 | 0.0120 | 0.0104 | 0.0120 |
| 8.8 | 0.0103 | 0.0106 | 0.0100 | 0.0103 |

Table S2: The standard deviation (StdDev) values of the fittings in the cumulant expansion analysis used in dynamic light scattering (DLS). The intensity-intensity time correlation function *G*^(2)^(*q*, *τ*) at each pH value was measured four times and StdDev 1, 2, 3 and 4 were obtained.
